# Cognitive performance in dialysis patients - "when is the right time to test?"

**DOI:** 10.1186/s12882-021-02333-x

**Published:** 2021-06-02

**Authors:** Hristos Karakizlis, Stefanie Thiele, Brandon Greene, Joachim Hoyer

**Affiliations:** 1 Department of Nephrology, Philipps-University of Marburg, Baldingerstrasse, 35033 Marburg, Germany; 2 Department of Nephrology, Justus-Liebig-University of Gießen, Klinikstrasse 33, Gießen, Germany; 3 Institution of med. Biometrics and Epidemiology, Philipps-University Marburg, Robert-Koch-Strasse, Marburg, Germany

**Keywords:** RBANS, Cognitive performance, Hemodialysis patients, Neurocognitive testing, Cognitive impairment

## Abstract

**Background:**

Cognitive impairment in chronic kidney disease, especially in end stage renal disease, is a public health problem. Nevertheless, the cause of chronic kidney disease still remains unclear. A prevalence of cognitive impairment in patients with end stage renal disease of up to 87% has been found.

**Methods:**

The study at hand deals with the research on the – potential – effect of timing on cognitive performance when testing cognitive impairment in hemodialysis patients during the dialysis cycle. We tested cognitive performance with a neuropsychological test battery (RBANS, Repeatable Battery for the Assessment of Neuropsychological Status) on two occasions while patients were on dialysis as well as on a dialysis-free day. In addition, all participants were rated using the Geriatric Depression Scale (GDS) and several demographic and clinical variables were recorded in order to investigate their possible influence on cognitive performance.

The patients were recruited in three dialysis centers in the central region of Hesse, Germany. Twenty-six participants completed the 3 testings during a period of 6 weeks. The testing was carried out in the dialysis centers.

**Results:**

Looking at the total scale score, patients achieved the best cognitive performance in the RBANS during the first 2 h on dialysis with 81.1 points. When comparing the scores of the three measurement occasions (first 2 h, Timepoint 1 vs. last 2 h, Timepoint 2 vs. dialysis free day, Timepoint 3, however, no significant difference in the total scale score was detected. But patients showed significantly better cognitive performance in language in the first 2 h (*p* < 0.001) as well as in the last 2 h (*p* < 0.001) compared with the dialysis-free day.

**Conclusion:**

Due to the high prevalence of cognitive impairment, there is an increasing need to assess cognitive function in dialysis patients. Our data show that the time point of testing (first 2 h on hemodialysis vs. last 2 h on hemodialysis vs. Hemodialysis free day) had no influence of cognitive function in hemodialysis patients in routine indications.

## Background

Cognitive impairment in chronic kidney disease (CKD), especially in end-stage-renal-disease (ESRD), has increasingly been researched on in the last years but the cause of CKD is still unknown and might be multifactorial. As the prevalence of cognitive impairment especially in patients with ESRD is up to 51–76% [1, 2], partly up to 87% [3] it is necessary and important to focus on both cause and diagnostic. Especially because hemodialysis patients need cognitive skills to understand and follow health-related information [4].

Several factors such as older age [5] and worsening kidney function itself [6–9] can cause cognitive impairment as well as uremia [10, 11] and cerebrovascular diseases [12]. We would like to emphasize that patients with CKD and especially ESRD have a higher risk of incident stroke [13–15] and have a higher prevalence of white matter lesions and silent brain infarcts [16, 17]. Further, white matter hyperintensity and ventricular and hippocampal atrophy is associated with cognitive impairment [18] and patients with a history of stroke or silent brain infarcts have an increased risk of dementia [16, 17, 19]. Also, the presence of depression can affect the results of cognitive performance [9, 20].. There are several test procedures for detecting depression, of which the Geriatric Depression Scale (GDS) is a test frequently used in clinical practice.

Low diastolic blood pressure in the elderly appears to be associated with an increased risk of developing dementia [21]. Intradialytic hypotension is a common phenomenon of hemodialysis patients and occurs in over 30% of patients and is not only associated with increased mortality [22] but also appears to have an impact on the cognitive performance of dialysis patients. The relationship between cognitive impairment and the reduction of cerebral blood flow has also been shown in recent retrospective and prospective studies [23, 24].

As hemodialysis patients are subject to acute hemodynamic changes and large fluid shifts during dialysis previous studies investigated that the point of testing cognitive impairment is relevant. Especially the dialysis itself causes acute deterioration to the patients compliance and could lead to not understanding medication plans when the doctor patient communication takes place during dialysis [25].

Since hemodialysis itself seems to affect cognitive function several studies investigated whether the time point of testing might lead to different results in cognitive functioning. Thus one previous study suggested that cognitive function was worse during dialysis and best the day after dialysis [25]. In addition to these findings further studies found that cognitive function seems to be best the day after dialysis in comparison shortly before dialysis [26, 27]. When focusing on the time directly after dialysis, however, the results differed from the findings of the other studies. While one study result shows an improvement in global neuropsychological functioning [28] other studies demonstrate that cognitive function gets worse after dialysis and the greatest cognitive impairment seems to be 67 h after dialysis [10]. In accordance with these findings individual cognitive fluctuations with mainly a decline in attention and executive function after dialysis were described [29]. Studies with a greater interval between their testing showed no difference in cognitive functioning [30].

Due to these various findings, we investigated whether the point of testing hemodialysis patients during a dialysis cycle in comparison to the day after dialysis has an effect on cognitive performance.

## Methods

### General data

We studied cognitive performance with a neuropsychological test battery (RBANS, Repeatable Battery for the Assessment of Neuropsychological Status) while patients were on dialysis as well as on a dialysis-free day. For better comparability the neuropsychological battery was administered three times: firstly, during the first 2 hours of dialysis (T1), secondly during the second 2 h of dialysis (T2) and thirdly on a dialysis-free day (T3). All three tests were carried out within a time interval of two weeks, respectively. The GDS was also performed once on all participants to assess the possible influence of depression.

The tests were conducted with a “standard dialysis population” in the administrative region of “Middle Hesse” at three separate outpatient dialysis centers. Criteria to be included in the study were: at least 18 years of age, native German speaker and at least 6 months on dialysis. Patients under the legal supervision of a guardian were excluded from the study. Recruitment was carried on until the desired number of 31 participants was reached.

This study was approved by the ethics committee of the University of Marburg and conforms to the Declaration of Helsinki. Written informed consent was obtained from of all participants.

### RBANS

The RBANS is a brief, individually-administered test measuring cognitive function across five domains: attention, language, visuospatial/constructional abilities, and immediate and delayed memory. Performance in each domain is measured by the subtests (12 in total) described below, which are combined to yield five scaled “index scores” gauging function in the respective domains. These five index scores are in turn combined to yield a composite score referred to as the “total scale score”. Stimuli are contained in a wire-bound, easel-type booklet, making the test easily portable and allowing for bedside administration. Total administration time is 20–30 min. Normative information from the manual for the index and total scores is based on 540 healthy adults who ranged in age from 20 to 89 years [31, 32].

The battery was designed to be amenable to the construction of multiple equivalent forms, and there are different, equivalent versions of the RBANS available for test-retest use.

The domains comprise the following subtests:

1. Immediate Memory

- List Learning: immediate recall of a 10-item list of words.
- Story Memory: a 12 item story, read aloud for immediate recall over two trials [32].

2. Visuospatial/Constructional

- Figure Copy: copying a geometric figure of 10 parts.
- Line Orientation: a 10 item line orientation test [32].

3. Language

- Picture Naming: 10-line drawings which the subject must name
- Semantic Fluency: the total number of examples generated for a given semantic category within 60 s [32].

4. Attention

- Digit Span: analogous to digits forward on the WAIS [33]. There are two strings of digits in each item, each pair increasing in length from 2 to 9 digits. The second string of a given length is only read if the first string is failed.
- Coding: numbers rather than symbols were chosen for the response in order to avoid the possible detrimental effect of a constructional apraxia on performance [32].

5. Delayed Memory

- List Recall: free recall of the words from the List Learning task.
- List Recognition: yes/no recognition testing for memory of the words from the List Learning task.
- Story Recall: free recall of the story from the story memory test.
- Figure Recall: free recall of the figure from the figure copy subtest [32].

All subtests of the RBANS were scored according to standardized age-adjusted criteria. Raw scores from the 12 subtests were converted to age- adjusted scaled scores before calculating an index score for each domain. The sum of the index scores was then converted to a total scale score.

### Tests and testing order

For each patient all three tests, including tests on the dialysis-free day, were conducted on site in the dialysis center. All testing was carried out by the same person who was trained prior to the commencement of the neuropsychological assessments.

Before testing we randomized the order of testing (T1, T2 and T3) for each patient to avoid possible confounding effects of the test scores due to learning effects. Thus, for each patient it was randomly chosen at which time point in the dialysis cycle the RBANS was administered to the individual for the first, second and third time. For every desired test time a different version of the RBANS was used: version A at T1, version B at T2 and version C at T3.

### Additional data

In addition to the RBANS, all participants were rated using the Geriatric Depression Scale and several demographic and clinical variables were recorded in order to investigate their possible influence on cognitive performance, such as age, sex, education, dialysis rhythm and duration, type of dialysis, years of dialysis, and possible nicotine, alcohol or drug abuse leading to renal disease. Independent mortality risk factors such as coronary heart disease, myocardial infarction, arterial hypertension, diabetes mellitus, stroke, dementia, depression as well as vital signs such as blood pressure and heart rate, and laboratory values, such as hemoglobin, albumin, pH, bicarbonate, cholesterol, creatinine, urea and Quality of Dialysis measured in Kt/V were also considered.

### Statistical analysis

For statistical computing we used “R program for statistical computing” [34]. We first looked at the measure of central tendency (mean, median, minimal and maximal value) and measure of variation (standard deviation) of the index and total scale score. For further analyses a linear mixed model was used for comparing the five cognitive domains across T1, T2 and T3. The domains are reflected in index scores which are summed in the total scale score. The within-patient correlation in the test scores has been taken into account. A similar mixed model was used to analyze if an increasing trend in patients’ scores with each additional repetition of the test has occurred.

## Results

### Sample characteristics and patients

Recruitment was carried out in three dialysis centers in the central Hessen region. Twenty-six patients completed all three testings at the predefined test points over a period of 6 weeks. Five patients had to be excluded, due to intercurrent diseases and in-hospital treatments.

The average age of the patients was 64.8 ± 14.4 years. Of these, 19 (73.1%) were male and 7 (26.9%) were female. All patients completed general school education for 9 years, 27% or 15% had school education for one- or three more-years equivalent to advanced high school education. 77% completed vocational training including community college and 15.4% completed academic education at a university. Characteristics and demographic data of the 26 patients are listed in Table 1.

**Table 1:** Demographics of the patients and etiology of renal diseases

Demographics	Patients (***n*** = 26) M (SD)
Age	64.8 (14.4)
Gender	Male 73.1%
School-Education	High-school 57.5%
	Senior-high 26.9%
	College 15.4%
Job-Education	Community college 76.9%
	University 15.4%
	None 7.7%
dialysis vintage (y)	5.65(4.39)
Kt/V	1.7 (0.3)
**Blood**	
Hemoglobin	11.5 g/dl (1.1)
Creatinine	9.46 mg/dl (2.6)
Albumin	38.2 mg/dl (4.9)
HCO3^−^	23.2 mmol/l (3.3)
**Clinical Data**	
Blood Pressure	127.8/68.1 mmHg (17.2/11)
Heart frequency	73/min (10.8)
**Etiology of renal disease**	
Diabetic Nephropathy	23.1%
Vascular/Hypertensive kidney disease	19.2%
Glomerulonephritis and systemic diseases	34.6%
Polycystic kidney disease	3.9%
Unclear	7.7%
Others	11.5%

*Kt/V* measurement of dialysis quality, *HCO3-* bicarbonate, *y* year

For additional information considering the underlying diseases leading to CKD, laboratory values were recorded. The mean values of our sample showed Hb 11.5 ± 1.1 g/dl, Creatinine 9.46 ± 2.6 mg/dl, albumin 38.2 ± 4.9 mg/dl, HCO3–23.2 ± 3.3 mmol/l, systolic and diastolic blood pressure of 127.8 ± 17.2 mmHg and 68.1 ± 11.0 mmHg respectively, pulse frequency 73 ± 10.8 /min and Kt/V 1.7 ± 0.3.

### Cognitive test results

**Fig. 1:**
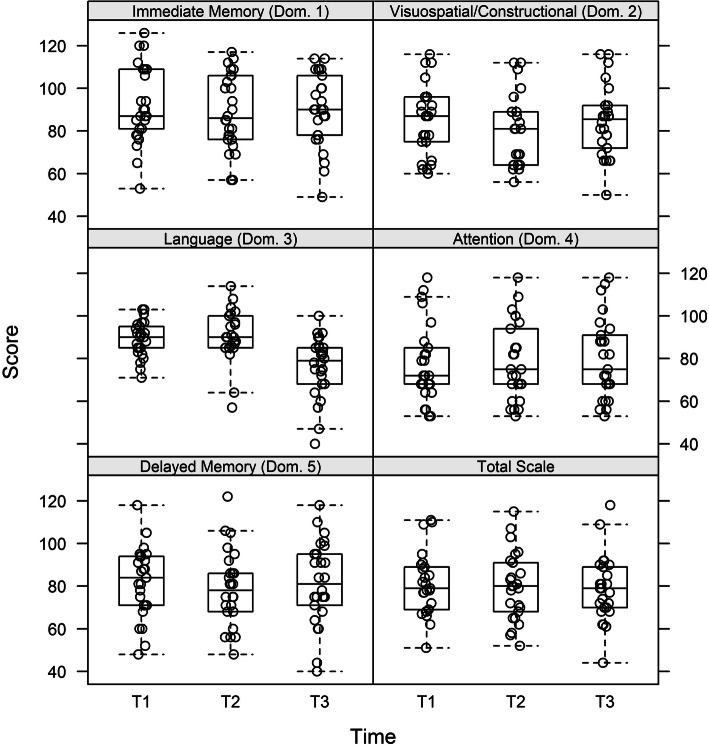
Index scores of each domain (Dom.) (1–5) and Total Score divided up to the time points. In the total scale there are no differences seen in the index scores. Also, in domain1,2,4 and 5 are no differences in cognitive performance in the individual test times occurred. Domain 3 (language) however, shows a significant difference from the time of testing T1 vs. T2 (*p* < 0.001) and test time of T1 vs. T3 (*p* < 0.001). T1 = timepoint 1, first 2 h on dialysis, T2 = timepoint 2, last 2 h on dialysis, T3 = timepoint 3, dialysis free day

First of all, we looked at the total scale score and the index scores of each individual domain compared across T1, T2 and T3 which are shown in Fig. 1. Comparing the three time points in time of testing (T1 vs. T2, T2 vs. T3 and T2 vs. T3) there was no statistically difference in the total scale score. The best cognitive performance was achieved by using the RBANS at T1 with 81.1 points. At T2 an average value of 79.6 and at T3 of 78.6 points was reached.

Also, by looking at the individual domains’ patients showed no significant difference in the scores at T1, T2 and T3 in domain immediate memory, visuospatial/constructional, attention and delayed memory.

Significantly different results were detected in domain 3 (language). Patients showed significantly better cognitive performance in language at both T1 (*p* < 0.001) and T2 (*p* < 0.001) compared with T3. In this domain, test results dropped from 76.5% at T3 to 89.1% at T1 and 90.8% at T2. These results clearly show that the reduction of the scores in domain 3 (language) concerns the entire patient group rather than being an effect of an individual deterioration from one of the patients results (see Fig. 1).

**Fig. 2:**
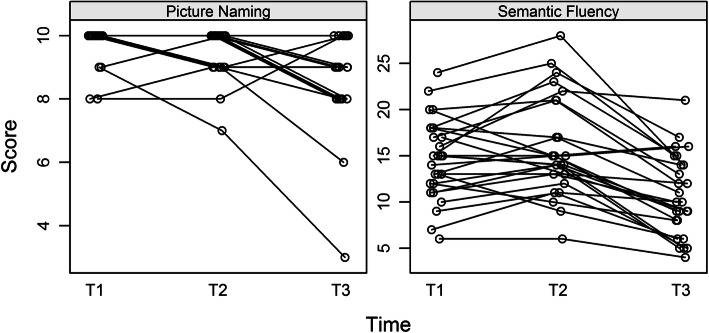
This figure shows the row-data of the two tests of domain 3 (language). There is a reduction in scores across the whole patients’ groups in both tests (Picture Naming and Semantic Fluency) at test-time-points. T1, T2 and T2. T1 = timepoint 1, first 2 h on dialysis, T2 = timepoint 2, last 2 h on dialysis, T3 = timepoint 3, dialysis free day

Domain 3 (language) comprising the picture-naming and semantic fluency subtests were further analyzed separately in order to investigate possible reasons for the significant differences in the scores. Therefore, raw scores were analyzed which showed that the deterioration at T3 was present in both of the subtests (see Fig. 2). In the picture naming subtest, the mean dropped from 9.8 at T1 and 9.6 at T2 respectively to 8.7 at T3 (all *p* < 0.001) and in the semantic fluency subtest from 14.7 at T1 and 15.8 at T2 to 10.7 at T3 (all *p* = < 0.001).

As seen before by looking at the index score there is again a reduction in all scores across the whole patient group and not a single poor test result by one of the patients at that time.

### Test repetition and learning effects

**Fig. 3:**
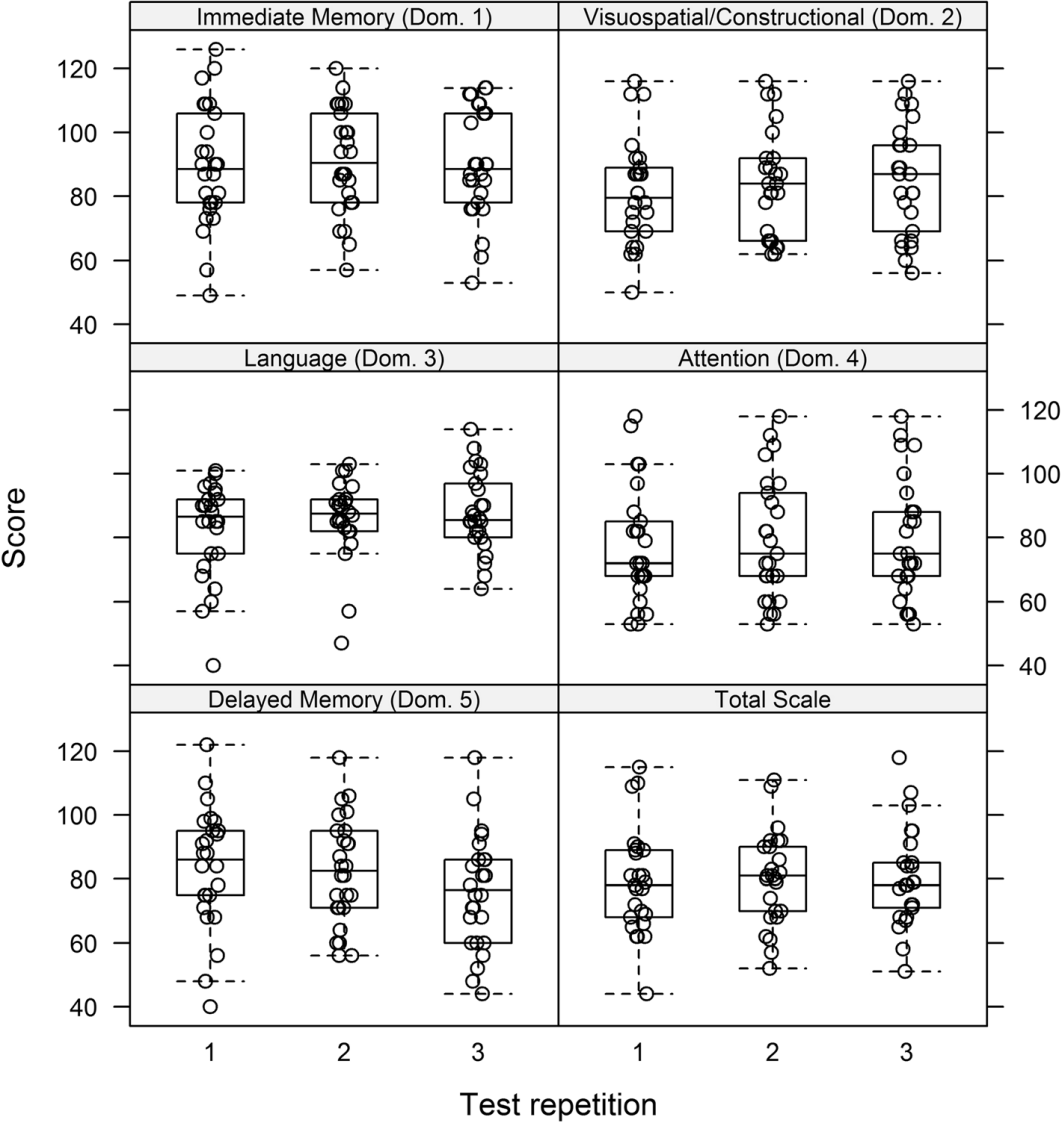
Index Scores of the domains (Dom.) 1 to 5 and Total scale score by number of test repetitions. No learning effect was seen during the various test-time points

We also analyzed the index scores and total scale score grouped by looking at the number of test repetitions i.e., if the participant was being administered the test for the first, second or third time. None of the index scores or the total scale score (*p* = 0.48) showed a noticeable trend. Thus, no significant learning effect was detected (see Fig. 3). Noteworthy is the significantly decreasing trend of average 3.8 points per repetition (*p* = 0.004) in domain 5 (delayed memory).

## Discussion

The aim of the current study was to evaluate the variability of cognitive performance in relation to the time of testing. The main question was if there is an optimal time of testing dialysis patients. This time should be used as a basis for future research in the field of cognitive impairment in dialysis patients. Neurocognitive testing was set in the first 2 h of dialysis, the last 2 h of dialysis and on dialysis free days. The results of our study indicate that the time point of testing cognitive function by using the RBANS in hemodialysis patients did not show any clinical impact.

No statistically measurable differences in cognitive performance in the overall results (total scale score) were detected. By looking at the individual domains poorer cognitive performance was seen in domain 3 (language) on the dialysis-free day (T3) in comparison to the two remaining points in time of testing during dialysis (T1 and T2).

In contrast to our results previous studies reported that the “optimal” time point for carrying out the test is 24 h after dialysis [10, 25]. However, dialysis treatments of the past that date back to the times of acetate dialysis [10] cannot be compared with nowadays modern treatments of dialysis with bicarbonate. Nausea and vomiting were extremely common among patients using these older methods, which could be a possible explanation why they achieved better test results on the dialysis-free days.

A study from 2007 using 30 dialysis patients demonstrated that the best cognitive performance was achieved on a dialysis-free day and that the first hours of dialysis were associated with significantly worse cognitive performance [25]. Here, a comprehensive test battery with an analysis of different cognitive domains was used, and especially in the domains “delayed verbal and visual immediate memory” a significantly impaired cognitive performance was seen during testing on dialysis. While language was not affected, a moderate difference in cognitive performance was found in the field of executive function [25]. A possible reason for the differing results in our study is that all patients were tested at the predefined test points over a period of 6 weeks, whereas in the study by Murray et al. the number of patients who did not complete all tests was greater (15 of 33 patients were not tested at all test sessions).

The use of different study designs could be another explanation for the differing results. In order to avoid possible learning effects, we left a time period of 14 days between every testing, selected a test battery for repeated measurements to be used at T1, T2 and T3 and randomized the sequence of test sessions T1, T2 and T3 for each patient.

In another study of Drew et al., cognitive performance was tested on a total of 40 patients just before dialysis and during the first hour after the beginning of the dialysis session. Their findings are consistent with our results, as there were no significant differences in cognitive performance between the time points of investigation [30]. However, in that study, a test on the dialysis-free day was not performed.

Williams et al. in 2004 reported a decline in cognitive performance in hemodialysis patients in auditory memory and in attention with the greatest impairment occurring 67 h after dialysis. However, they did not perform their testing during dialysis, but 1, 24 and 67 h after dialysis [10]. In our study, we did not see any overall deterioration in cognitive performance at the different points of testing, surprisingly we did see a deterioration in cognitive performance in the domain of language at test point T3. In the study of Williams et al., only two subtests showed a change in cognitive performance. The reason for this does not appear to be entirely clear right now. Since the deteriorations occur on dialysis-free days we do not believe that hemodynamic fluctuations are responsible for this. The accumulation of toxins on the non-dialysis day would rather be responsible for the cognitive changes. However, further studies are needed to finally answer this question.

In a cross-sectional observation study of 47 hemodialysis patients in 2014, the majority of patients examined did not show significant individual cognitive variation. The minority of patients who showed a fluctuation in cognitive performance showed a deterioration in attention and executive function after dialysis [29]. This is in accordance to our findings.

In accordance with these findings an improvement of cognitive impairment has also been found after dialysis in global neuropsychological functioning by using a computer based assessment battery [28]. We did not focus on the time after dialysis but the results, and that the majority of patients being tested after dialysis had a stable performance actually confirms the findings of our study.

One recent study also tested the cognitive performance in hemodialysis patients on dialysis and the day after dialysis. The authors hypothesized that a single dialysis improves cognitive function in hemodialysis patients. They showed an improved performance in logical and visual long-term memories after the dialysis session [27]. But, as in our study, neither the performance in short-term and working memories nor in verbal fluency and planning behavior was changed. An almost similar study design with testing before dialysis and the day after with an improvement of cognitive function after hemodialysis especially in attention was described by Griva et al. [26]. The different findings might be explained with the difference in age of the patients. Age is a risk factor for cognitive decline and in both studies patients were approximately 10 years younger than in our sample [26, 27]. Dasgupta et al. report that cognitive function is significantly reduced during hemodialysis treatment in the majority of patients [2]. The main difference between the study design and our study is that the timing of the test was significantly different from ours. We tested during the first 2 h and the last 2 h of dialysis and on the dialysis-free day at 14-day intervals, whereas Dasgupta et al. tested immediately before dialysis and at the end of dialysis and 1 week later shortly before dialysis. Testing on the dialysis-free days did not take place. The differences in the results between the studies appears to be related to the timing of the post-dialysis testing.

A recent study showed a clear relationship between cerebral blood flow and cognitive performance. Patients in the study were tested on dialysis and also on dialysis-free days and showed a significant deterioration in executive function during dialysis treatment [24]. The significant differences between those results and our study results could be explained by the number of hypertensive patients. In our study population there were fewer hypertensive patients which could be less sensitive to blood pressure fluctuations.

One study reported that cognitive performance in hemodialysis patients not only depends in the time of testing but also on the testing environment. As the best cognitive performance was achieved by testing before hemodialysis in a separate room the authors suggest that a standardization test should be used before hemodialysis in a separate room [35]. Bearing in mind that previous studies tested patients in their dialyzing room might partly explain the decline in cognitive performance on dialysis. We are currently unable to confirm this hypothesis as patients in our study were also tested during dialysis in their dialysis room and had no decline in cognitive impairment on dialysis compared to the dialysis free day.

Independent of the time of testing, the study participants showed a high prevalence of cognitive impairment. In accordance with previous findings our results show a decline in cognitive performance in executive functions, language, attention and delayed memory as well as immediate memory [25, 36–38]. It should be mentioned that adhering to the strict scoring criteria of the figure copy and figure recall test might lead to lower raw scores in the visuospatial/constructional and delayed memory subtest [39]. We strictly observed these scoring criteria as the purpose of this study was to compare the results of the different time points without classifying the severity of cognitive impairment.

A potential cause for the high prevalence of cognitive impairment in hemodialysis patients might be a high prevalence of stroke, white matter lesions and silent brain infarcts [17, 24]. As previously determined, there is a high prevalence of cerebral atrophy in hemodialysis patients [17, 40]. Recent studies complement the findings by describing a significant correlation between age and whole brain atrophy as well as hippocampal atrophy and hyperhomocysteinemia [41].

In a further study Harciarek et al. investigated the semantic and phonemic fluency performance in hemodialysis patients and determined a decline in phonetic fluency while patients showed a stable performance in semantic fluency [42]. The stable performance in semantic fluency was also seen in other studies [27, 30] but cannot be confirmed in our study. A possible explanation might be that the missing improvement in semantic fluency seen in the results of Schneider et al. [27] reflect the cognitive decline seen in our result on the dialysis free day.

A consideration of Drew at el. especially on executive function and memory over years in hemodialysis patients showed a decline in executive but not memory function. Age was a strong risk factor for cognitive decline for the executive function [43].

The missing deterioration in memory function, in contrast to our study, might be caused by learning effects. By testing a validated test battery for repeated testing with different test versions we can exclude learning effects and this could be an explanation for the different results.

The current study may be limited by:

- our small sample size and
- patients, who already noticed cognitive impairment themselves, might have rejected from participating in our study.
- the study population of 65 years is slightly younger than the average dialysis patient, which could also have an effect on cognitive performance.
- an overrepresentation of male subjects in our study.

Due to the sample size and the slightly younger age of the study population as well as and the overrepresentation of male subject, the results may not be transferable to the general dialysis collective. Additionally, it must be noted that the tests were conducted in German and therefore the results may not be generalizable to all demographic groups. It is also possible that there were still residual confounders, since possible influencing factors may not yet be known and could not be taken into account.

Nevertheless, the study also has its strengths and advantages:

- First of all, it is a prospective randomized study. In order to exclude learning effects in the individual test points, three different versions of the tests were conducted in each case.
- The second important strength is the implementation of an extensive neurocognitive test battery, which is more sensitive than screening tests in detecting mild cognitive impairment. The cognitive battery used in this study (RBANS) incorporates multiple previously validated cognitive tests and includes different versions. These versions are validated for showing an absence of learning effects when retesting [32]. Therefore, learning effects can be excluded.
- Third, almost all patients included in our study completed the tests on all three predefined testing points.
- Additionally, patients who participated in the study form an average of dialysis patient in terms of education, medical history, gender and underlying disease.

## Conclusion

Our study shows that the time point of testing (first 2 h on hemodialysis vs. last 2 h on hemodialysis vs. Hemodialysis free day) had no influence on cognitive function in hemodialysis patients in routine indications. Cognitive tests can thus be used as an element in routine checkup on dialysis patients during the visit. This provides us with an overview of the cognitive function of the patients and can also be used as a course parameter to detect a possible deterioration of the cognitive function at an early stage.

## Data Availability

The datasets used or analyzed during the current study are available from the corresponding author on reasonable request.

## Abbreviations

CKD: Chronic kidney disease
Dom: Domain
ESRD: end stage renal disease
GDS: Geriatric Depression Scale
RBANS: Repeatable Battery for the Assessment of Neuropsychological Status
Hb: Hemoglobin
HCO3-: bicarbonate
Kt/V: measurement of Dialysis Quality
T1: Timepoint 1, first 2 h on dialysis
T2: Timipoint 2, last 2 h on dialysis
T3: Timepoint 3, dialysis free day
WAIS: Wechsler adult intelligence scale
